# Naturally produced opsonizing antibodies restrict the survival of *Mycobacterium tuberculosis* in human macrophages by augmenting phagosome maturation

**DOI:** 10.1098/rsob.150171

**Published:** 2015-12-16

**Authors:** Shashi Kant Kumar, Padam Singh, Sudhir Sinha

**Affiliations:** 1 Division of Biochemistry, CSIR-Central Drug Research Institute, Sector-10, Jankipuram Extension, Lucknow 226031, India; 2 Academy of Scientific and Innovative Research, New Delhi, India

**Keywords:** *Mycobacterium tuberculosis*, latent tuberculosis infection, antibody opsonization, phagosome maturation

## Abstract

This study investigated the hypothesis that serum antibodies against *Mycobacterium tuberculosis* present in naturally infected healthy subjects of a tuberculosis (TB) endemic area could create and/or sustain the latent form of infection. All five apparently healthy Indian donors showed high titres of serum antibodies against *M. tuberculosis* cell membrane antigens, including lipoarabinomannan and alpha crystallin. Uptake and killing of bacilli by the donor macrophages was significantly enhanced following their opsonization with antibody-rich, heat-inactivated autologous sera. However, the capability to opsonize was apparent for antibodies against some and not other antigens. High-content cell imaging of infected macrophages revealed significantly enhanced colocalization of the phagosome maturation marker LAMP-1, though not of calmodulin, with antibody-opsonized compared with unopsonized *M. tuberculosis*. Key enablers of macrophage microbicidal action—proinflammatory cytokines (IFN-*γ* and IL-6), phagosome acidification, inducible NO synthase and nitric oxide—were also significantly enhanced following antibody opsonization. Interestingly, heat-killed *M. tuberculosis* also elevated these mediators to the levels comparable to, if not higher than, opsonized *M. tuberculosis*. Results of the study support the emerging view that an efficacious vaccine against TB should, apart from targeting cell-mediated immunity, also generate ‘protective’ antibodies.

## 1. Introduction

The prospects for tuberculosis (TB) control remain uncertain with increasing incidences of treatment failure and diminishing options for a safe and effective chemotherapy, particularly against drug-resistant *Mycobacterium tuberculosis* (Mtb). This alarming situation has compelled researchers to revisit the scope for a therapeutic or prophylactic vaccine. However, despite the cell-mediated immunity (CMI) playing a key role in protection against TB, BCG and other vaccines targeting CMI have performed dismally [1] suggesting that contributions from other components of the immune system could be crucial. An early adaptive immune response towards Mtb serves to restrict the infection to an asymptomatic state known as ‘latent TB infection (LTBI)’. Effectiveness of this response can be gauged from the fact that even though a third of the world population is considered to be infected with Mtb, less than 10% of the infected persons develop TB during their lifetime [2,3]. An insight into the mechanism(s) underlying the creation and perpetuation of LTBI may therefore unravel the components of the immune system that confer protection against TB and pave the way for a more efficacious vaccine.

Several lines of evidence suggest that B cells could have an important role in immunity against Mtb. Lymphoid follicles peripheral to the granuloma in lungs of TB patients have been described as the sites for priming of immune cells [4]. These follicles comprise aggregates of B cells interspersed with macrophages harbouring Mtb and T cells located in close contact with the B-cell foci. Essentially similar observations were reported in studies performed on the mouse [5] and non-human primate [6] models of TB. In the latter study, B-cell (plasma cell) clusters within the granuloma were found to actively secrete antibodies (Abs) to Mtb antigens. In two independent studies, B-cell deficient mice have shown greater susceptibility to the infection [7,8].

While B cells may contribute to CMI by presenting antigens to T cells or by secreting cytokines, Abs produced by them can also modulate the immune response. The role of opsonizing Abs in protection against Mtb infection in mice has been highlighted in a number of studies. Opsonizing IgG monoclonal Abs (mAbs) to the Mtb cell surface component lipoarabinomannan (LAM) prolong the survival of infected mice [9]. A human IgA mAb to the Mtb cell membrane protein alpha crystallin (Acr) has been shown to reduce bacterial load in mice transgenic for human Fc*α*R1 receptor [10]. Opsonization with rabbit or human (produced by injecting BCG to the donors) Abs to LAM has also been shown to promote, respectively, the killing of Mtb [11] or BCG [12] by human macrophages. Nonetheless, the mechanism by which Abs confer protection against intracellular infections largely remains obscure.

Phagosome acidification through fusion with lysosomes is critical for killing of the bacilli within macrophages and its inhibition is a major survival mechanism employed by Mtb [13]. Acidification can directly restrict microbial growth as well as activate the lytic enzymes (e.g. cathepsins) within the mature phagosomes [14]. Calmodulin, which is a mediator of [Ca^2+^]-dependent signalling, plays an important role in phagosome maturation [15,16]. The lysosome-associated membrane proteins (LAMPs), present abundantly on mature phagosomes, are also considered to be essential for microbicidal activity [17]. In addition, proinflammatory cytokines such as IFN-*γ* promote phagosome maturation and acidification through acquisition of LAMP-1 and vacuolar ATPase [18]. Phagosomes containing viable Mtb lack v-ATPase [19], LAMP, cathepsin D [20,21] and Rab7 [22]. A role for nitric oxide (NO), produced by inducible NO synthase (iNOS), in the control of TB is indicated by the observation that NO can kill Mtb within human macrophages [23]. Importance of iNOS is also evident from exacerbation of infection in mice following inhibition of the enzyme [24] or inactivation of the corresponding gene [25]. Expression of iNOS has been reported in alveolar macrophages or broncho-alveolar lavage fluid of TB patients [26]. For its survival, Mtb impairs the production of NO by inhibiting iNOS recruitment onto phagosomes [27,28].

In a TB-endemic country such as India, which accounts for nearly a quarter of global burden of the disease, almost the entire population is considered infected with Mtb [2]. Based on T-cell reactivity to Mtb-specific antigens ESAT-6 and CFP-10, nearly 90% of urban Indians were found to be latently infected with TB [29]. In a related study, high levels (up to 96% positivity) of Abs to ESAT-6 and CFP-10 were seen in the sera of persons with LTBI, particularly those belonging to a TB endemic area [30]. Our own earlier study [31] has noted that 100% sera of apparently healthy north Indian subjects contain high titre Abs to the membrane-associated antigens of Mtb. A recent study has elaborated that membrane-associated proteins of Mtb are indeed preferred targets for Ab response during LTBI [32].

With an aim to determine whether and how the naturally occurring Abs against Mtb limit the infection in a TB endemic setting, we have previously reported that opsonization with Ab-rich heat-inactivated autologous sera enhances killing of the bacilli by macrophages of apparently healthy Indian subjects [31]. Presently, we have explored the possible mechanism underlying this enhanced killing. Specifically, we looked for modulation of the key mediators of phagosome maturation, LAMP-1 and calmodulin, and key mediators of intraphagosomal killing, vacuole acidification and iNOS/NO expression. In this endeavour, we have strived to quantify in a robust and unbiased manner the changes occurring at the level of an ‘average’ infected host cell by performing high-content imaging of a suitably large number of monocyte-derived macrophages from each donor.

## 2. Material and methods

### 2.1. Materials

Mtb H37Ra was obtained from ATCC (ATCC no. 25177). RPMI 1640 medium, FITC-conjugated anti-human IgG antibody, Lysotracker Red, SYTO 9 and anti-rabbit Alexa Fluor antibody were from Invitrogen. Rabbit anti-iNOS and anti-LAMP-1 antibodies were from Thermo Pierce Biotech. Anti-CD14 mAb was from Boehringer-Mannheim GmbH. Rabbit anti-sheep RBC antibody was from Rockland Immunochemicals. mAbs against Mtb-Acr (IT-4) and LAM (CS-35) were gifted by UNDP/World Bank/WHO Special Programme for Research and Training in Tropical Diseases. Affinity purified peroxidase-conjugated anti-human and anti-mouse IgG antibodies, anti-calmodulin antibody, o-phenylenediamine (OPD), diaminobenzidine (DAB), 2 µM amine-modified green fluorescent polystyrene beads, citrate phosphate dextrose and other laboratory chemicals were from Sigma-Aldrich. BD optEIA cytokine ELISA kits and Middlebrook (MB) media and supplements were obtained from BD Biosciences. Fluorescent NO probe FL2E was purchased from Strem Chemicals, Inc. Ficoll-isopaque was obtained from GE-Biosciences. Ninety-six-well clear glass bottom black imaging plates were from Thermo Nunc.

### 2.2. Donors

All subjects were BCG vaccinated four to six weeks after birth and, being residents of a TB endemic region (north India), were considered as environmentally exposed to Mtb. None of them had any past or present clinical, radiological or bacteriological sign or symptom of TB. Serum samples were collected from donors' blood and aliquots were stored at −80°C. Heat inactivation of the sera, where ever indicated, was done by incubating an aliquot at 56°C for 1 h in a water bath.

### 2.3. Mycobacteria

Three-week-old culture of Mtb H37Ra on Lowenstein–Jensen (L–J) medium was harvested and washed with OADC supplemented MB 7H9 broth containing 0.05% Tween-80 (MB medium). One hundred milligrams of wet bacterial pellet was suspended in 5 ml MB medium containing 15% glycerol and subjected to 20 pulses (10 s each) of bath sonication to disperse clumps. This procedure was earlier found optimal for de-clumping of bacilli without any loss of viability [31]. The suspension was centrifuged lightly (1000 r.p.m. × 5 min) to settle the residual clumps and supernatant, containing mostly single bacilli as determined by Zeihl–Neelsen (Z–N) acid-fast staining, was divided into aliquots and was stored at −80°C. Colony forming units (cfu) were counted on OADC supplemented MB 7H11 agar plates.

Heat-killed Mtb was prepared by incubating an aliquot at 90°C for 30 min in a water bath, and killing was confirmed by the absence of viable colonies on MB 7H11 agar plates.

For fluorescent labelling, aliquots of bacilli were thawed and centrifuged (8000 r.p.m. × 15 min). The pellet was re-suspended in 200 nM SYTO 9 green fluorescent stain (in carbonate buffer, pH 9.5). After incubation (37°C, 15 min), stained bacilli were collected by centrifugation and washed with PBS. Single-cell suspension of the fluorescent bacilli was prepared by probe sonication (10 s pulse) and the viability was determined by cfu assay.

### 2.4. *Mycobacterium tuberculosis* antigens

Mtb membrane and cytosol were prepared as described previously [31]. In brief, bacilli were washed and suspended in buffer containing protease inhibitors and probe-sonicated. The sonicate was centrifuged at 23 000*g* to remove unbroken cells and cell wall debris and supernatant was re-centrifuged at 150 000*g* to obtain the cell membrane (sediment) and cytosol (supernatant). Protein was estimated using the modified Lowry's method [33]. Antigens were sterilized by either autoclaving (membrane) or filtration (cytosol) through a 0.22 µM filter and stored at −80°C in aliquots.

### 2.5. Opsonization of *Mycobacterium tuberculosis* and isolation of opsonizing antibodies

Normal or fluorescent-labelled bacilli (5 × 10^7^ cfu) were incubated (37°C, 1 h, in a water bath) with heat-inactivated human serum (100 µl). Post-opsonization, bacilli were washed with PBS and suspended in RPMI1640 medium. To confirm whether they got opsonized with IgG antibodies, samples of unopsonized and opsonized non-fluorescent bacilli were applied on glass coverslips, dried, covered with blocking solution (3% BSA in PBS containing 0.05% Tween-20; PBS-T) and incubated (1 h) at room temperature (RT). After removing the blocking solution, bacilli were covered with FITC-conjugated rabbit anti-human IgG antibody (1 : 500) and re-incubated (1 h, RT). After washing with PBS-T, fluorescent images were acquired on a microscope. Single-cell suspensions of unopsonized or opsonized bacteria were prepared by probe sonication (10 s pulse) and their viability was checked by cfu assay.

For isolation of opsonizing antibodies, washed oposonized bacilli (non-fluorescent) were re-suspended in 0.5 ml elution buffer (0.2 M glycine-HCl, 0.2 M NaCl in water, pH 2.8). The suspension was incubated (30 min, RT) with continuous mixing and centrifuged (12 000 r.p.m. × 15 min) to sediment the bacilli. Supernatant containing eluted Abs was collected and its pH was adjusted to 7.2 using 1 M Tris.

### 2.6. Complement assay

Inactivation of complement in heat-inactivated sera was confirmed by sheep red blood cell (SRBC) haemolysis assay [34]*.* In brief, SRBCs were sensitized with rabbit anti-SRBC antibody (working dilution was predetermined by SRBC agglutination assay) in HEPES buffer. Antibody-sensitized SRBCs were incubated (37°C, 30 min) with dilutions of normal or heat-inactivated serum in gelatin-HEPES buffer in a round bottom microtitre plate. The plate was centrifuged and supernatants transferred to another plate. OD was taken at 540 nm and fractional haemolytic values were calculated.

### 2.7. ELISA

Serum antibody titres against membrane and cytosolic antigens of Mtb were determined by ELISA. Antigens (10 µg protein ml^−1^ in 0.05 M carbonate buffer, pH 9.5) were coated on ELISA plates (50 µl well^−1^, overnight, 4°C) and uncoated surfaces were blocked (2 h, RT) with blocking solution (2% skimmed milk powder in Tris-buffered saline containing 0.05% Tween-20, TBS-T). Test sera (diluted in blocking solution) were added to the antigen- or buffer-coated wells (50 µl well^−1^) and incubated (90 min, RT). After washing with TBS-T, peroxidase-conjugated anti-human IgG Ab (1 : 4000) was added to the wells (50 µl well^−1^) and the plate was incubated again (90 min, RT). To the washed plates, substrate (OPD) solution was added (50 µl well^−1^) and incubated (20 min) in dark. The reaction was stopped by adding 7% H_2_SO_4_ and OD was read at 492 nm in a plate reader. Differences in mean OD of the antigen- and buffer-coated wells were calculated for each serum dilution and expressed as ΔOD.

Antibodies to Mtb LAM and Acr were detected by competition ELISA as follows. The plate was coated with Mtb membrane antigens and test sera, either alone or mixed (1 : 1) with anti-Acr (IT-4) or anti-LAM (CS-35) mAbs, were added to the plate (total volume 50 µl well^−1^). Final serum dilutions were 1 : 200, 1 : 400 and 1 : 800 and final mAb dilutions were 1 : 1000 (IT-4) and 1 : 100 (CS-35). The plate was incubated (90 min, RT) and, after washing, re-incubated with peroxidase-conjugated anti-human IgG Ab (90 min, RT). The remaining steps (development of colour and OD reading) were as above.

### 2.8. Immunoblotting

The profile of Mtb membrane antigens recognized by donor sera was visualized by immunoblotting. Mtb membrane proteins were resolved by SDS-PAGE (12.5% gel) using a wide sample well and electroblotted onto nitrocellulose membrane. Later, blot was cut into vertical strips and individual strips were blocked with blocking solution (as for ELISA) for 2 h at RT. Strips were then incubated (90 min, RT) with the test sera (diluted in blocking solution). After washing, affinity purified peroxidase-conjugated anti-human IgG antibody (1 : 4000) was added to the strips and incubated (90 min, RT). One strip was incubated overnight (at 4°C) with anti-LAM mAb (CS-35, 1 : 50) and probed with affinity purified proxidase-conjugated anti-mouse IgG antibody (1 : 4000). After final washing, antigen bands were detected by the peroxidase substrate DAB.

### 2.9. Macrophages

Peripheral blood mononuclear cells were isolated from citrated blood by centrifugation over Ficoll-isopaque*.* Cells were suspended in RPMI medium supplemented with 10% heat-inactivated pooled normal human serum (complete medium) and dispensed in either 96-well black plates with clear glass bottom (4 × 10^5^ cells well^−1^) or 48-well normal culture plates (7 × 10^5^ cells well^−1^). The plates were incubated overnight in a CO_2_ incubator and wells were washed thoroughly with plain RPMI medium to remove non-adherent cells. By counting the adherent cells per field of the microscope, 5–7% of the input cells were determined as adherent. More than 98% of adherent cells were viable by Trypan Blue assay, and more than 98% of them were considered as monocytes by fluorescent immunostaining for cell surface marker CD14 (electronic supplementary material, figure S1A). Prior to experiments, the macrophages were allowed to differentiate in complete RPMI medium for 7 days in the CO_2_ incubator.

### 2.10. Assay for intracellular multiplication of *Mycobacterium tuberculosis*

Macrophages in 48-well culture plates were washed with plain (without serum) RPMI medium. Bacilli (unopsonized/opsonized), suspended in the plain RPMI medium, were added to the respective wells at a MOI (multiplicity of infection) of 5 bacilli cell^−1^ [31]. Some wells were left uninfected as control. After 3 h of incubation (5% CO_2_, 37°C), cells were washed thoroughly with plain RPMI medium to remove extracellular bacilli and complete RPMI medium was added to each well. Cells in respective wells were lysed on day-0 (immediately after 3 h of infection) and day-5 with 0.1% saponin. cfu assays in lysates were performed on MB 7H11 agar plates.

On day-5, prior to cell lysis, supernatants were collected from control and infected wells, filtered with 0.2 µm filters, divided into aliquots and stored at −80°C for cytokine assays.

### 2.11. Cytokine assays

Estimations of IFN-*γ*, IL-6 and IL-10 in culture supernatants were performed by using corresponding ELISA kits (BD OptEIA).

### 2.12. Staining for LAMP-1, calmodulin and inducible NO synthase

Macrophages in 96-well glass bottom plates were washed with plain RPMI medium and allowed to internalize (3 h, 37°C, 5% CO_2_) the heat-killed, unopsonized or opsonized fluorescent bacilli or green fluorescent polystyrene beads (MOI = 5 particles cell^−1^). Later, cells were washed thoroughly with plain medium to remove extracellular bacilli or beads. Cells were then fixed (4% paraformaldehyde in PBS, 15 min, RT), washed, permeabilized (0.2% Triton X-100, 15 min, RT) and blocked with 3% BSA (in PBS-Tween). Primary antibodies (diluted in blocking solution, 1 : 500 for anti-LAMP-1 and anti-iNOS; and 1 : 200 for anti-calmodulin antibodies) were added to respective wells and incubated (1 h, RT). After washing with PBS-Tween, cells were again incubated (1 h, RT) with Alexa Fluor 647-conjugated anti-rabbit IgG antibody (1 : 1000). Cells were washed with PBS-Tween and nuclear staining was performed with Hoechst 33342.

### 2.13. Staining for acidic compartments

After the 3 h uptake of bacilli or beads (described above), macrophages were washed thoroughly with plain RPMI1640 medium to remove extracellular particles. To label the intracellular acidified compartments, cells were pulsed (1 h, 37°C, 5% CO_2_) with 100 nM Lysotracker Red DND-99 in plain medium. Cells were then washed with PBS and fixed. After another washing with PBS-Tween, nuclei were stained with Hoechst 33342.

### 2.14. Staining for intracellular nitric oxide

Macrophages in 96-well glass bottom plates were allowed to internalize the non-fluorescent bacilli (heat killed/unopsonized/opsonized). Cell-trappable fluorescent NO probe Cu-FL2E, prepared according to the supplier's protocol, was added to the wells (1 µM, 100 µl well^−1^) and incubated (12 h, 37°C, 5% CO_2_). After removing the supernatant, cells were washed with PBS and nuclear staining was performed using Hoechst 33342.

### 2.15. Image acquisition and analysis

Automated fluorescent imaging was performed on a Thermo-Cellomics Arrayscan VTi High-Content Imaging system equipped with metal-halide light source, Hamamatsu ORCA-R2 camera and Zeiss Plan-neofluar 40× (0.75 NA) and 20× (0.45 NA) objectives. Triple colour epi-fluorescent images were acquired using XF-93 Hoechst (nucleus), XF-100 FITC-GFP (fluorescent bacilli/bead, NO probe Cu-FL2E), XF-53 Texas Red (Lysotracker Red) and XF-110 Cy5-sensitive (Alexa Fluor 647) filters (Omega Optical, Brattleboro, VT, USA) of the Arrayscan. The microscope was set to autofocus after each field.

Images of nearly 500 cells (50 fields) in an experimental set-up were acquired with 40× objective using Cellomics Compartmental Analysis Bioapplication software (v. 3.1) and stored in Cellomics Image Database Server for further analysis. For each cell, three images were acquired: one for the cell nucleus (channel-1), one for the fluorescent bead/bacilli containing compartment (channel-2) and one for the LysoTracker Red/Alexa Fluor positive compartment (channel-3). The acquisition parameters were set so that the LysoTracker Red/Alexa Fluor signal was minimal in the uninfected (control) cells. All images in each experimental set-up were acquired and analysed in a fully automated and unbiased manner.

To measure the intensity of Lysotracker Red/Alexa Fluor-labelled proteins and number of particles (bacilli/beads) in individual cells, images were analysed using Cellomics Compartmental Analysis Bioapplication. As summarized in the electronic supplementary material, figure S2, nuclei (channel-1) were identified using an object identification algorithm based on intensity thresholds between adjacent pixels, segmented and used for the quantification of cell number and to define spatial location of individual cells. Identified objects in a field were subsequently selected or rejected on the basis of shape, size and intensity. To delineate cell boundary, concentric Ring and Circ regions were defined in channels-2 and -3, respectively, at 35 pixels distance from nuclear boundary (selected object). Spots in channels 2 (bead/bacilli) and 3 (Lysotracker Red/Alexa Fluor) were identified using fixed threshold method and segmented. Ring spot count (number of bacilli/bead) and Circ spot total intensity (Lysotracker Red/Alexa Fluor-labelled protein intensity) parameters were measured within target region (Ring and Circ) of each cell. Numerical values of these parameters for individual cells were imported to Sigma Plot data sheets and used for statistical analyses.

For the analysis of colocalization of fluorescent bacilli (heat killed, unopsonized or opsonized) with Lysotracker Red/Alexa Fluor, stored images were re-scanned using Cellomics Colocalization Analysis Bioapplication. Object identification parameters in all channels were set as described above. As shown in the electronic supplementary material, figure S2, regions of interest (ROI) were defined in channel-2 at a distance of 8 pixels from the identified bacilli. Within ROI, channels 2 (bacilli) and 3 (Lysotracker Red/Alexa Fluor) were selected as target channels and Mander's colocalization coefficient (MC) [35] was determined for each cell using ‘overlap coefficient’ parameter of Colocalization Analysis Bioapplication. Numerical values of MC were imported to SigmaPlot data sheets and used for statistical analyses.

To measure intensity of Cu-FL2E-labelled intracellular NO, images of nearly 100 cells were acquired (with 20×, 0.45 NA objective) and analysed using Cellomics Compartmental Analysis Bioapplication. For each cell, two images were acquired: one for cell nucleus (channel-1) and the other for fluorescent probe Cu-FL2E (channel-2). Object identification in both channels was performed as described above (electronic supplementary material, figure S2). The Circ region was defined at around 28 pixel distance from the nuclear boundary in channel-2 to delineate the cell boundary. Circ spot total intensity (Cu-FL2E intensity) for each cell was measured and numerical values were transferred to SigmaPlot data sheets for statistical analysis.

Representative images were imported from Image Database server and, to make them presentable, uniform brightness/contrast was adjusted using Adobe Photoshop. 7.0 software.

### 2.16. Statistical analysis

Statistical analyses were performed using Microsoft Excel and SigmaPlot. Significance level (*p* < 0.05) of differences was determined by either two-tailed Student's *t*-test or Wilcoxon rank-sum test (for box plots).

## 3. Results

### 3.1. Donor sera contained high titres of antibodies against *Mycobacterium tuberculosis* cell envelope antigens including lipoarabinomannan and alpha crystallin

**Figure 1.**
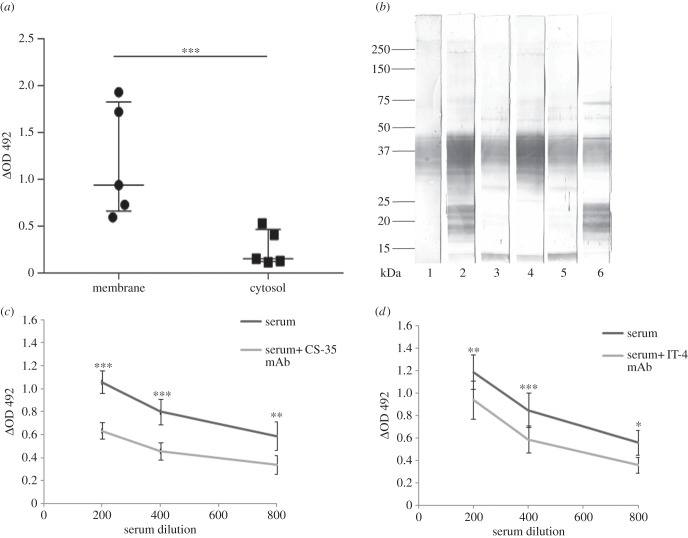
Profile of anti-Mtb antibodies in the donor sera. Panel (*a*) shows Ab titres against membrane and cytosolic antigens, determined by ELISA. Median, interquartile range and individual OD values of five donor sera (diluted 1 : 400) are shown. Panel (*b*) depicts immunoblotting of membrane antigens with the mAb CS-35 showing presence of LAM (lane 1) and with donor sera (lanes 2–6) showing Abs to various antigens including the characteristic band of LAM. Panels (*c*) and (*d*) show, respectively, competitive binding of serum Abs (mean OD ± s.e. of five sera) to the membrane antigens LAM and Acr in presence of mAbs CS-35 and IT-4. *p < 0.05, **p < 0.02, ***p < 0.01.

It has previously been observed by us [31] and others [30] that serum Abs against Mtb antigens could serve as more reliable markers for LTBI than the PPD skin test, cytokine assay or a chest radiogram. Sera from all, apparently healthy, donors of this study exhibited high titres of Abs against membrane-associated antigens of Mtb (median OD at 1 : 400 dilution = 0.938, figure 1*a*). In comparison, Ab titres against cytosolic antigens were significantly lower (median OD = 0.154, *p* < 0.01). These results are consistent with our previous observations on a diverse set of healthy donors from the same endemic area [31].

As some studies [9–11] had shown that opsonization with, or passive transfer of, Abs against Mtb cell surface antigens LAM and Acr provide protection against the infection, we also looked for these Abs in the sera of our donors. The characteristic diffused LAM band of Mtb cell membrane was recognized by all sera (figure 1*b*). Another band of approximately 14 kDa (possibly Acr) was also stained by all sera, though with a variable intensity. Presence of LAM and Acr in Mtb membrane was confirmed by their reactivity with corresponding mAbs (electronic supplementary material, figure S1B and S1C). Both mAbs also inhibited significantly the binding of serum Abs to the membrane (figure 1*c*,*d*) indicating presence of anti-LAM and anti-Acr Abs in the sera. Apart from these, certain other antigens were also recognized by the individual donor sera.

Altogether, these results support the view that healthy residents of a TB endemic area carry circulating Abs against cell envelope antigens of Mtb [32], even though there could be individual variations in their antigen specificities.

### 3.2. Opsonization of *Mycobacterium tuberculosis* with antibodies present in heat-inactivated sera enhanced phagocytosis, intracellular killing and proinflammatory cytokine production by the donor macrophages

Heat inactivation of sera depleted the complement without affecting Ab titres (electronic supplementary material, figure S1D and S1E). Complement depletion was determined as the loss of haemolytic activity, in contrast to a concentration-dependent activity in the normal (unheated) serum. Opsonization of Mtb bacilli with the IgG class of Abs present in heat-inactivated donor sera was confirmed by staining with fluorescent anti-human IgG Ab (electronic supplementary material, figure S1F). A comparison of the profile of membrane antigens recognized by the pooled donor serum with those recognized by opsonizing Abs of the same serum revealed that not all Abs were involved in opsonization. Abs to at least two major antigens (of approx. 48 and 80 kDa) were absent from the opsonizing Ab extract (electronic supplementary material, figure S1G). It was also confirmed, by performing cfu assays, that opsonization did not result in loss of bacterial cell viability (electronic supplementary material, figure S1H). Similarly, consistent with an earlier report [36], fluorescent (SYTO 9) labelling of the bacilli also did not result in loss of viability.

**Figure 2.**
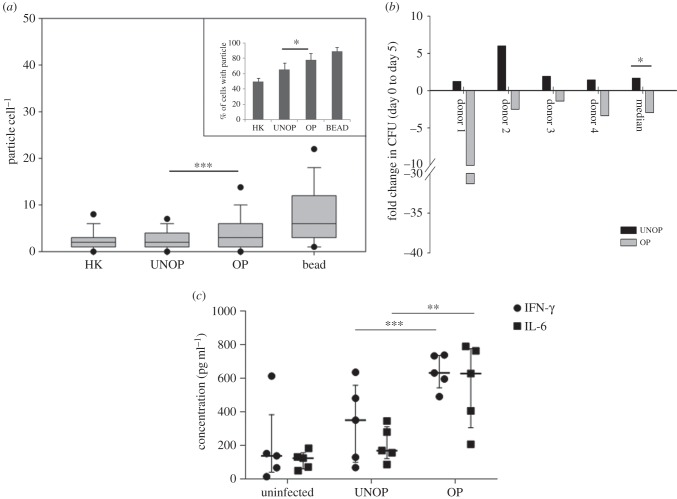
Particle uptake, intracellular killing of Mtb and production of proinflammatory cytokines by donor macrophages. Panel (*a*) shows number of fluorescent particles (HK, heat-killed Mtb; UNOP, unopsonized Mtb; OP, opsonized with heat-inactivated autologous sera; bead, polystyrene beads) per cell and (inset) % cells with at least one particle after 3 h of internalization. The box and whisker plot represents median and 5th–95th percentile values for five donors and bar diagram (inset) represents mean ± s.e. values of pooled data from 500 cells (100 from each of the five donors). Panel (*b*) shows fold change (between days 0 and 5) in viable counts (cfu) of UNOP and OP Mtb within the macrophages from four donors. Individual as well as median values are represented by the bar graph. Panel (*c*) shows IFN-*γ* and IL-6 production (median, interquartile range and individual values) by the uninfected and infected macrophages of five donors. Significance of differences between OP and UNOP clusters (indicated by horizontal lines) were: *p < 0.05, **p < 0.02, ***p < 0.01.

To determine the effect of opsonization with Abs present in heat-inactivated sera on the uptake of Mtb by macrophages, fluorescent-labelled unopsonized, opsonized or heat-killed bacilli were internalized into monocyte-derived macrophages from all five donors. Fluorescent polystyrene beads were used as control and enumeration of intracellular particles (bacilli/beads) was done by fluorescent particle counting during high-content image analysis. Figure 2*a* shows the proportion of cells with at least one particle as well as the number of particles per cell. Compared with the unopsonized, opsonized bacilli infected a significantly larger proportion of cells (65.2 ± 8.7% and 77.6 ± 8.5%, respectively, *p* < 0.05). Corresponding values were lowest for heat-killed bacilli (49.6 ± 4.6%) and highest for beads (88.8 ± 5.4%). Moreover, a significantly larger number of opsonized (than unopsonized) bacilli entered each infected cell (respective median values = 3 and 2, *p* < 0.01). Corresponding values for heat-killed Mtb and beads were 2 and 6. Thus, opsonization resulted in infecting a higher proportion of macrophages, each with a greater number of bacilli.

To determine the ability of macrophages to restrict intracellular multiplication of Mtb, viable bacilli (cfu) were counted on days 0 (immediately after 3 h internalization) and 5 post-infection in four of the donors. Whereas host cells allowed up to sixfold (median, 1.7-fold) multiplication of the unopsonized bacilli (figure 2*b*), they significantly curtailed (*p* = 0.029) the survival of opsonized bacilli (median—2.9-fold). In fact, day 5 cfus were lower than day 0 cfus in all cases with opsonized Mtb, suggesting killing, to a variable extent, of the initial uptake.

The proinflammatory cytokines IFN-*γ* and IL-6 play an important role in induction of the microbicidal mechanism of macrophages [37,38]. Concentrations of both were found to be significantly higher (median of five donors = 630 and 640 pg ml^−1^, respectively, figure 2*c*) in culture supernatants of macrophages infected with Ab-opsonized than unopsonized bacilli (median = 350 and 175 pg ml^−1^, respectively). Production of IL-10, which is an anti-inflammatory cytokine [39], however did not differ significantly (data not shown).

### 3.3. Opsonization of *Mycobacterium tuberculosis* with serum antibodies facilitated the phagosome maturation

We next determined the effect of serum Ab opsonization of Mtb on expression and localization of the key mediators of phagosome maturation—LAMP-1 and calmodulin—within the donor macrophages.

**Figure 3.**
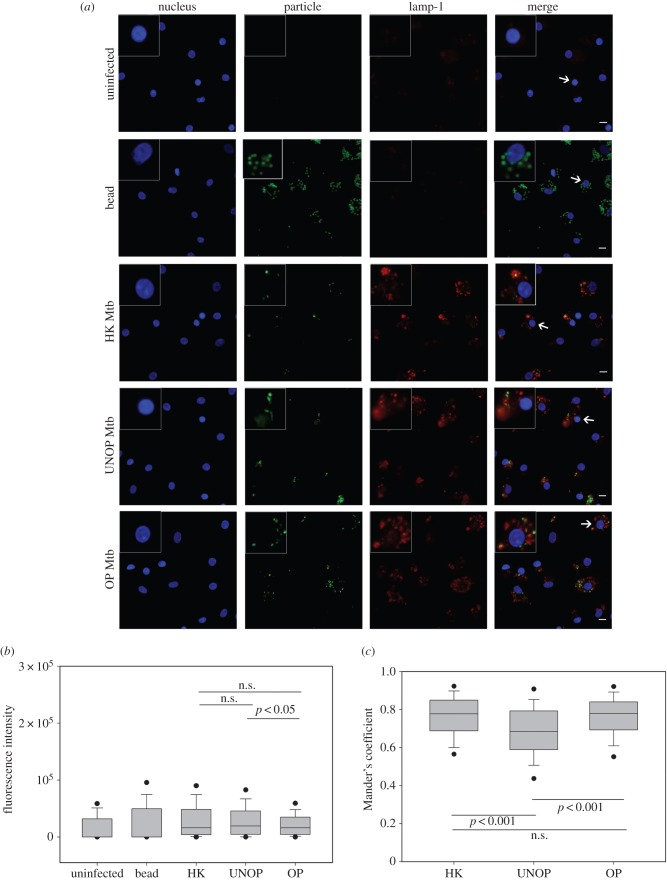
Opsonization with serum Abs enhanced the colocalization of Mtb with LAMP-1. Macrophages from all five donors were allowed to internalize beads or Mtb (HK/UNOP/OP) followed by detection of LAMP-1 by immunofluorescence. Panel (*a*) shows representative images (nuclei stained with Hoechst 33342) taken with 40× objective, including enlarged view of one cell (indicated by arrows in merged images) displayed in the insets. Scale bar (shown in merged images) is 10 µM. Panel (*b*) shows whole-cell intensity of LAMP-1 under different experimental conditions. Box and whisker plot (median and 5th–95th percentile values) represents pooled data of 500 cells (100 cells from each donor, each cell with at least one particle). Panel (*c*) shows degree of colocalization of LAMP-1 with Mtb measured as Mander's coefficient. The box and whisker plot (median and 5th–95th percentile values) represents pooled data of 250 cells (50 cells from each donor, each cell with at least one bacillus). Significance of differences (*p*-values) are shown above or below the corresponding pair of boxes in panels (*b*) and (*c*) (n.s., not significant, *p* > 0.05).

Figure 3 depicts the levels of expression and colocalization of LAMP-1 with Mtb (unopsonized/opsonized/heat killed). The representative photomicrographs (figure 3*a*) show approximately 10 nuclei/40× objective field (average from 50 fields), indicating that there was enough space around the seeded macrophages for adherence and spread. At the used MOI (5 particles cell^−1^), most cells exhibited phagocytosis without getting overcrowded with the particles (column 2). The uninfected control cells did not stain for LAMP-1 (column 3). Likewise, the cells bearing polystyrene beads (used as another control) also did not express LAMP-1 despite the fact that the beads were phagocytosed more avidly than the bacilli (figure 2*a*). In contrast to controls, punctated LAMP-1 protein was prominently visible in the perinuclear cytoplasmic space of cells bearing Mtb (column 3). Furthermore, the protein appeared to be mostly colocalized with the bacilli (column 4).

In concordance with the visual impression, fluorescence intensity (FI) of LAMP-1 in Mtb (all three preparations) bearing cells was significantly higher than that in the uninfected or bead-bearing cells (median FI = 0, figure 3*b*). However, within the Mtb-bearing cell populations, FI of those with opsonized bacilli (median FI of five donors = 16 067) was significantly lower (*p* = 0.029) than of those with the unopsonized bacilli (median FI = 19 323). Conversely, colocalization of LAMP-1 with opsonized Mtb, measured as MC (figure 3*c*), was significantly higher (median MC = 0.78) than that with the unopsonized bacilli (median MC = 0.68, *p* < 0.001). Thus even though cells bearing opsonized (compared with unopsonized) Mtb contained a lesser amount of LAMP-1, a greater amount of it actually got colocalized with the bacilli.

**Figure 4.**
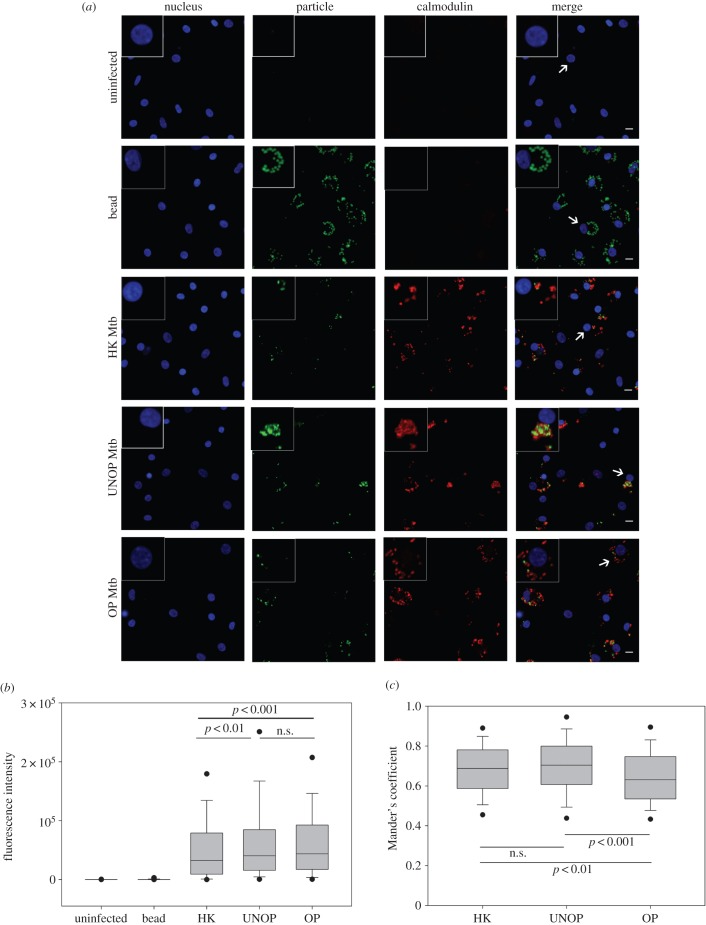
Opsonization with serum Abs reduced the colocalization of Mtb with calmodulin. Cells were allowed to internalize bead or Mtb (HK/UNOP/OP) and calmodulin was detected by immunofluorescence. Panel (*a*) shows representative images (taken with 40× objective) including enlarged view of one cell (indicated by arrows) displayed in the insets. Scale bar is 10 µM. Panel (*b*) shows whole-cell intensity of calmodulin under various conditions. The box and whisker plot (median and 5th–95th percentile values) represents pooled data of 500 cells (100 cells from each donor, each cell with at least one particle). Panel (*c*) shows colocalization of calmodulin with Mtb, measured as Mander's coefficient. The box and whisker plot represents median and 5th–95th percentile values from pooled data of 250 cells (50 cells from each donor, each cell with at least one bacillus). The *p*-values are also depicted in panels (*b*) and (*c*).

The pattern of calmodulin expression (figure 4) was also similar to LAMP-1, with the control cells (uninfected or bead-bearing) showing no detectable staining for the protein (figure 4*a*). On the other hand, calmodulin was expressed specifically and prominently by the cells containing Mtb (all preparations) and appeared to be colocalized with the bacilli. In tune with this observation, FI of calmodulin in cells carrying Mtb was significantly higher than both the controls (median FI = 0, figure 4*b*). However, there was no difference in the FI of cells that were infected with either opsonized (median FI of 5 donors = 43 752) or unopsonized (median FI = 40 304) Mtb. Interestingly, in contrast to the observation with LAMP-1, colocalization of calmodulin with opsonized Mtb (median MC = 0.631, figure 4*c*) was significantly less (*p* < 0.001) than that with the unopsonized bacilli (median MC = 0.704).

### 3.4. Opsonization of *Mycobacterium tuberculosis* with serum antibodies enhanced the microbicidal capability of donor macrophages

To further probe the mechanism underlying enhanced intracellular killing of serum Ab-opsonized Mtb, we quantified phagosome acidification as well as expression of iNOS/NO, which are considered to be the key mediators of macrophage microbicidal action.

**Figure 5.**
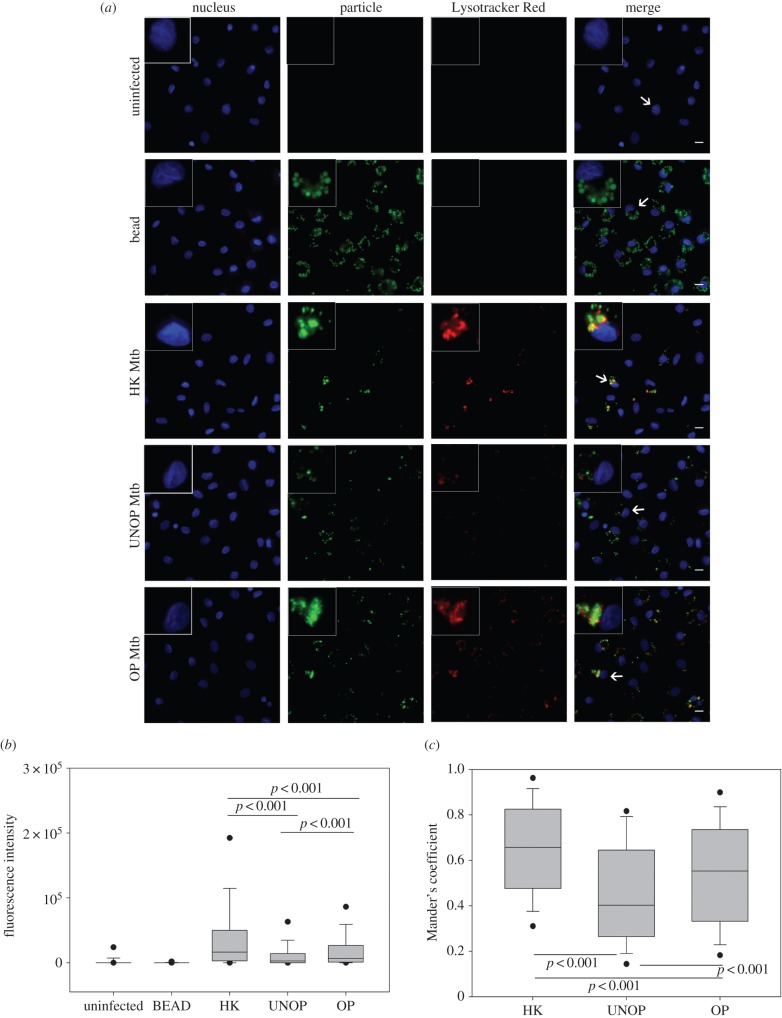
Opsonization with serum Abs enhanced colocalization of Mtb with Lysotracker Red. Cells were allowed to internalize bead or Mtb (HK/UNOP/OP) followed by labelling of acidified compartments with Lysotracker Red. Panel (*a*) shows representative images (with 40× objective), including enlarged view of one cell (indicated by arrows) displayed in the insets. Scale bar is 10 µM. Panel (*b*) shows whole-cell intensity of Lysotracker Red under different conditions. Box and whisker plot (median and 5th–95th percentile values) represents pooled data of 500 cells (100 cells from each donor, each cell with at least one particle). Panel (*b*) shows colocalization of Lysotracker Red with the bacilli, measured as Mander's coefficient. The box and whisker plot represents median and 5th–95th percentile values from pooled data of 250 cells (50 cells from each donor, each cell with at least one bacillus). The *p*-values are also depicted in panels (*b*) and (*c*).

FI of Lysotracker Red and its colocalization with Mtb served as an indicator for phagosome acidification (figure 5). The representative photomicrographs (figure 5*a*) show that acidification occurred specifically and prominently (with punctate appearance) in cells which had phagocytosed Mtb and not in controls (uninfected or bead-bearing cells). Within the Mtb-bearing cell populations, a visibly lesser acidification was noted in the case of unopsonized as compared to opsonized or heat-killed bacilli (column 3). Moreover, colocalization of Lysotracker Red with unopsonized bacilli also appeared to be less than that with the other two preparations (column 4).

In agreement with the microscopic observations, FI of Lysotracker Red was significantly higher (*p* < 0.001) in cells bearing the opsonized (median FI of five donors = 6831, figure 5*b*) than unopsonized (median FI = 2860) Mtb. Similarly, colocalization of the dye with opsonized bacilli (median MC = 0.55, figure 5*c*) was also significantly higher (*p* < 0.001) than that with the unopsonized ones (median MC = 0.40). These results indicate that phagocytosis of opsonized (compared with unopsonized) Mtb produced a significantly enhanced acidification of phagosomes.

**Figure 6.**
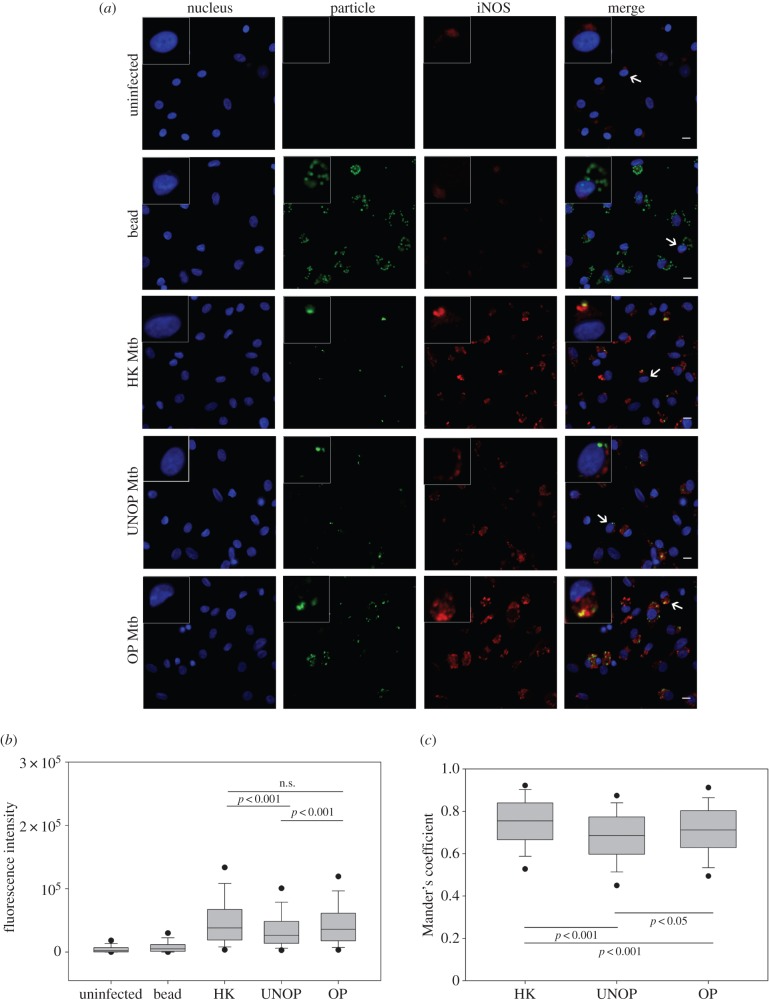
Opsonization with serum Abs enhanced colocalization of Mtb with iNOS. Cells were allowed to internalize fluorescent beads or Mtb (HK/UNOP/OP) and iNOS was detected by immunofluorescence. Panel (*a*) shows representative images (with 40× objective), including enlarged view of one cell (indicated by arrows) in the insets. Scale bar is 10 µM. Panel (*b*) shows whole-cell intensity of iNOS under different conditions. The box and whisker plot (median and 5th–95th percentile values) represents pooled data of 500 cells (100 cells from each donor, each cell with at least one particle). Panel (*c*) shows degree of recruitment of iNOS on bacilli measured as Mander's coefficient. The box and whisker plot represents median and 5th–95th percentile values of pooled data of 250 cells (50 cells from each donor, each cell with at least one bacillus). The *p*-values are also depicted in panels (*b*) and (*c*). (n.s., not significant, *p* > 0.05.)

Figure 6 depicts the expression as well as colocalization of iNOS. The photomicrographs (figure 6*a*) show that, apart from its high levels in Mtb-bearing cells, basal levels of the protein were also present in controls (with somewhat better expression in bead-bearing cells). Within the cells that harboured Mtb, a weaker expression as well as colocalization of iNOS was noted in the case of unopsonized as compared to the opsonized or heat-killed bacilli. Confirming these microscopic observations, expression (median FI of 5 donors = 36044, figure 6*b*) as well as colocalization (median MC = 0.71, figure 6*c*) of iNOS was also found to be significantly higher (*p* < 0.001 and 0.02, respectively) in cells containing the opsonized (compared to unopsonized) bacilli. Enhanced colocalization of iNOS with the opsonized Mtb is a particularly important observation since NO must be produced in the vicinity of the bacilli for its optimal microbicidal action [28].

**Figure 7.**
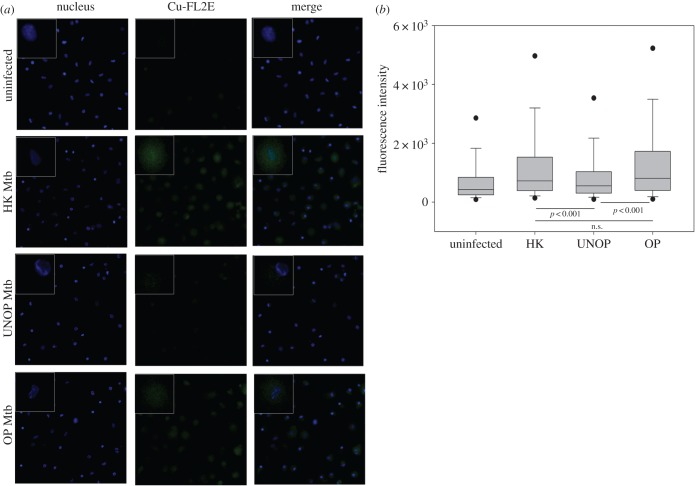
Opsonization of Mtb with serum Abs enhanced the production of NO. After internalization of Mtb (HK/UNOP/OP, non-fluorescent), cells were incubated for 12 h with 1 µM NO probe Cu-FL2E. Panel (*a*) shows representative images (taken with 20× objective), including enlarged view of one of the cells in the insets. Panel (*b*) shows whole-cell intensity of NO probe under different conditions. The box and whisker plot represents median and 5th–95th percentile values from pooled data of 300 cells (100 cells each from three donors). *p*-values are also shown in panel (*b*). (n.s., not significant, *p* > 0.05.)

Figure 7 depicts the detection and quantification of intracellular NO. The used cell-trappable fluorescent probe (copper complex of FL2E) allows direct imaging of NO produced by iNOS (as well as cNOS) in the living cells [40]. However, unlike the localized and punctated appearance of iNOS protein (figure 6*a*), the NO probe fluoresced in a ‘diffused’ manner (owing most probably to the gaseous nature of NO) in cells that harboured Mtb (figure 7*a*). Consistent with the iNOS expression levels, NO was also produced minimally in cells with unopsonized Mtb as compared to those with the opsonized or heat-killed bacilli. In agreement with the microscopic observations, FI of the NO probe was also significantly higher (*p* < 0.001) in cells with opsonized (median FI of three donors = 809) than those with unopsonized (median FI = 551) bacilli (figure 7*b*).

Altogether, these results indicate that opsonization with naturally produced serum Abs significantly enhanced the microbicidal potency of donor macrophages through increased levels of phagosome acidification as well as iNOS and NO production.

### 3.5. Extent of phagosome activation induced by heat-killed *Mycobacterium tuberculosis* was comparable with serum antibody opsonized *Mycobacterium tuberculosis*

The comparison of host cell responses to Ab-opsonized and heat-killed Mtb revealed some interesting results. Like the opsonized bacilli, heat-killed Mtb also induced a significantly enhanced colocalization of LAMP-1 (median MC = 0.77, figure 3) and expression as well as colocalization of Lysotracker Red (median FI = 16 384, MC = 0.65; figure 5), as compared with the unopsonized bacilli. Similarly, iNOS expression and colocalization (FI = 38 268, MC = 0.75; figure 6) and NO production (FI = 719, figure 7) were also significantly higher in the case of heat-killed Mtb. In fact, phagosome activation induced by the heat-killed Mtb, in terms of recruitments of Lysotracker Red (figure 5) and iNOS (figure 6), was even better than the Ab-opsonized bacilli. Colocalization of calmodulin with heat-killed Mtb, though similar to the unopsonized bacilli, was also significantly higher than Ab-opsonized bacilli (figure 4). These observations indicate that the quantum of phagosome maturation and activation induced by the heat-killed Mtb were comparable, if not better, than that induced by Ab-opsonized bacilli.

## 4. Discussion

We probed the hypothesis that antibodies against *M. tuberculosis* present in apparently healthy subjects of a TB endemic area could create and perpetuate the state of ‘LTBI’, thereby providing protection against active disease. All donor sera contained high titres of Abs against cell envelope antigens of Mtb, consistent with our own [31] and others' [30,32] prior reports. In the sera, we also detected Abs to two such antigens LAM and Acr that have particularly been linked with protection against the infection [9,10]. Post-natal vaccination of our donors with BCG is unlikely to have modified their Ab responses. In a study of healthy PPD negative children and young adults from India and England, all sera were found to contain Abs against Mtb antigens (higher titres in the case of Indians) even prior to vaccination [41]. More importantly, Ab titres remained unaltered when retested eight weeks after the BCG vaccination. This particular study also corroborates our observation that antigen specificity of naturally produced Abs could vary between individuals. Even though certain other studies have demonstrated occurrence of anti-mycobacterium Abs following BCG inoculation, they used higher inocula (over three times the vaccine dose) with [12] or without [42] a booster.

While allowing multiplication of the unopsonized bacilli, monocyte-derived macrophages of the donors killed, to a variable extent, Mtb opsonized with the Abs present in heat-inactivated autologous sera. In our previous study also, performed on a different set of donors from the same endemic area, we had shown a reduction in intracellular cfu following opsonization of Mtb (H37Rv) with serum Abs [31]. In a related study, de Vallière *et al.* [12] showed enhanced uptake and killing of BCG by donor macrophages following opsonization with heat-inactivated sera containing Abs against LAM. Abs in this case were produced by injecting two doses of BCG to the donors. Malik *et al.* [11] have also studied the fate of Mtb inside monocyte-derived macrophages from healthy donors after opsonizing the bacilli with Abs against LAM produced in rabbit. In this case, whereas Ab opsonization promoted bacterial killing, opsonization with human serum complement enhanced their survival which was attributed to a reduced [Ca^2+^] flux within macrophages.

The microbial cargo destined for degradation resides initially in early phagosomes which, through a series of membrane fusion and fission events, mature into late phagosomes or phagolysosomes [14]. We quantified, through high-content cell imaging and analysis, the effect of serum Ab opsonization on certain key mediators of phagosome maturation and activation. For this purpose, colocalization of the chosen events was determined by Mander's coefficient [35] which is based on Pearson's correlation coefficient and is widely used as a metric for colocalization in biological microscopy [43]. Importantly, MC is insensitive to signal intensity of fluorophores and measures actual overlap of the signals, and hence represents a true degree of colocalization. By comparison, the visual methods based on a ‘subjective’ identification of presence or absence of combined colour of two fluorophores provide only a qualitative assessment of colocalization.

The late phagosomes and lysosomes share overlapping molecular content, including LAMPs whose presence signifies successful completion of phagosome–lysosome fusion [14]. LAMPs are also considered essential for acquisition of microbicidal functions by phagosomes [17]. A significantly reduced colocalization of LAMP-1 on the unopsonized, compared with heat-killed, Mtb seen by us is consistent with an earlier report [20]. Using immunoelectron microscopy, the authors were able to show that phagosomes of human monocyte-derived macrophages harbouring heat-killed Mtb stained more intensely for LAMPs than those having live bacilli. However, of greater significance is our observation that serum Ab opsonization of the live bacilli enhanced this colocalization to a level closer to that attained by the heat-killed Mtb.

Certain proinflammatory cytokines have been shown to promote phagosome maturation. For instance, IFN-*γ* promotes mycobacterial killing by inducing phagosome–lysosome fusion and acidification through acquisition of LAMP-1 and vacuolar ATPase [18]. IL-6 specifically induces expression of Rab5 in macrophages [44] which coordinates membrane localization and activation of Vps34, leading to local synthesis of PI(3)P. In turn, PI(3)P facilitates downstream steps in phagosome maturation [14]. Consistent with our [31] and others' [45] prior observations, macrophages harbouring Ab-opsonized Mtb produced significantly higher concentrations of both the cytokines. Besides, Mtb is also known to induce production of the anti-inflammatory cytokine IL-10 which can impair phagosome maturation [39]. However, we did not observe any significant variations in IL-10 production.

Calmodulin, as a mediator of [Ca^2+^]-dependent signalling, also plays an important role in phagosome maturation [15]. Its role, however, is limited to the ‘tethering’ step which precedes docking of lysosomes onto phagosomes [16]. In our study, though macrophages with Ab-opsonized or unopsonized bacilli contained comparable amounts of calmodulin, its colocalization was significantly less in the case of opsonized bacilli. This could partly be explained by the fact that calmodulin is needed only transiently during fusion, and phagosomes containing IgG opsonized particles fuse rapidly with lysosomes [46]. Binding of IgG opsonized particles to Fc receptors creates ‘waves’ of [Ca^2+^] within the cell [47] and some studies have concluded that the elevated [Ca^2+^] levels are also required for fusion of lysosomes with Mtb-bearing phagosomes [11]. Nonetheless, the role of [Ca^2+^] in maturation of phagosomes or intracellular fate of pathogens remains controversial [48].

Acidification is the hallmark of a ‘functional’ phagosome. After interacting with discrete endosomal compartments, the phagosome becomes markedly acidic, highly oxidative, and gets enriched with hydrolytic enzymes that ultimately degrade its cargo [14]. In addition, the proton gradient across the phagosome membrane can restrict the microbial growth by stimulating natural resistance-associated macrophage protein-1. Induction of phagosome acidification by Ab-opsonized Mtb was evident from a significantly enhanced colocalozation of Lysotracker Red with the opsonized compared with unopsonized bacilli. Armstrong & D'Arcy Hart [13], using mouse macrophages and rabbit Abs against BCG, also showed enhanced acidification of phagosomes containing Ab-opsonized Mtb. However, in their study, intracellular killing of Mtb was not seen, due perhaps to an interplay of other factors such as the use of heterologous immune serum or presence of active complement in the serum.

iNOS is important for the control of Mtb infection, as evidenced by exacerbation of infection in mice following inhibition of the enzyme [24] or inactivation of the gene [25]. iNOS catalyses the synthesis of NO through oxidation of l-arginine on the cytosolic face of the phagosome membrane. NO thus synthesized subsequently diffuses into phagosomes where it can react with the microbe's respiratory chain and block electron transport as well as ATP biosynthesis [14]. We saw a significantly enhanced cellular expression as well as colocalization of iNOS with opsonized rather than unopsonized Mtb. Consistent with this, production of intracellular NO was also significantly enhanced in cells bearing the opsonized bacilli. Interestingly, the ‘inefficient’ bactericidal activity of iNOS/NO has been held responsible for driving the bacteria towards latency. Garbe *et al.* [49] have shown that the reactive nitrogen intermediates (RNIs) may push Mtb towards a stationary phase of growth. This finding is particularly relevant for TB granuloma where, despite the local presence of iNOS and NO [50], latent bacilli persist.

Phagocytosis of heat-killed Mtb also promoted phagosome maturation and activation, as evident from enhanced recruitments of LAMP-1, calmodulin and iNOS, acidification of phagosomes and production of NO. With respect to phagosome acidification and iNOS recruitment, the heat-killed bacilli performed even better than the Ab-opsonized ones. Some prior studies have also reported that, in contrast to live Mtb, phagosomes containing heat-killed bacilli fuse more efficiently with lysosomes [51]. Several cell envelope constituents of Mtb are known to interfere with macrophage signalling pathways. These include LAM and SapM which keep the bacilli in a PI(3)P-free environment [52], the secreted protein PtpA which disrupts the interaction between the HOPS complex and vacuolar ATPase to prevent phagosome acidification [53], and the cell wall protein LpqH which inhibits antigen processing [54]. Certain other cell wall components such as diacyltrehalose and sulfoglycolipid can also interfere with phagosome maturation [55]. Mtb AnsA causes asparagine deamination and supports mycobacterial growth through release of ammonia which elevates phagosomal pH [56]. Heat-killed Mtb is likely to be devoid of such ‘inhibitory’ molecules as they are mostly heat-labile. Compared with the live, the heat-killed Mtb bacilli have shown a significantly reduced amount of LAM on their surface [57].

Our observation that not all Abs present in the donor sera took part in opsonization precludes the possibility that the ‘non-immune’ serum IgG could also opsonize the bacilli indiscriminately. This inference remains valid even if one considered that some of the serum Abs (e.g. against approx. 48 and approx. 80 kDa antigens) did not bind to the Mtb cell surface merely because the corresponding antigens were occluded. Though we have not ventured to do so in this study, it would be interesting to learn if the 48 and 80 kDa antigens are indeed the major serologically active antigens MTB48 and MTB81 [58]. In the study by Armstrong & D'Arcy Hart [13], Mtb bacilli were incubated with non-immune as well as immune sera, washed and used for infecting mouse macrophages. Phagosome acidification was evident only with bacilli that were opsonized with Abs present in the immune serum. In a related study [59], opsonization of *Legionella pneumophila* with Abs in the immune serum was required to target the bacteria for lysosomal degradation and opsonization with non-immune serum did not produce such a consequence. Nonetheless, it may have been desirable to include in our study a group of unexposed (to Mtb) donors as ‘control’. Given the worldwide prevalence of LTBI [2,3], the possibility of accessing such donors is however quite remote. Implications of using an avirulent (H37Ra) rather than virulent (H37Rv) strain of Mtb in this study needs also to be considered. Genetically both the strains are not far apart [60] and there is hardly any difference in the way they interact with macrophages. At an infection ratio of one bacillus/cell, the average division times of both strains in human monocyte-derived macrophages were nearly equal [61]. The recognition of cell wall-associated ManLAM of both strains by mannose receptors restricts the phagosome–lysosome fusion to the same extent [57], and both the strains have an overlapping immunoproteome [62].

The most probable effector mechanism for Ab-mediated immunity is uptake of the opsonized pathogen through Fc gamma receptors (FcgR). FcgR–Ab interaction has been shown to enhance generation of reactive oxygen species (ROS) and RNI [63,64], as well as intracellular killing of the microbes [59]. On the other hand, FcgR knockout mice are highly susceptible to Mtb [65]. Apart from triggering the FcgR-mediated proinflammatory signals, it is also possible that the opsonizing Abs remain attached to bacterial surfaces even after phagocytosis to influence phagosome remodelling. In a recent study, *Salmonella typhimurium* was found to carry Abs into the cell and the cytosolic Ab receptor TRIM21 colocalized with the Ab-bound bacteria to elicit a proinflammatory response [66].

In the final analysis, opsonization of Mtb with naturally produced Abs may be able to contain but not eradicate the infection. This raises the obvious question as to whether one can enhance the effectiveness of Ab opsonization. Based on earlier reports [67], it can be hypothesized that specificity, source and nature of opsonizing Abs play an important role in intracellular killing of Mtb. We too have observed in this study that not all Abs present in the sera took part in opsonization. It has been observed that antibodies to cell surface antigens of Mtb produced by TB patients with active disease are of a low avidity and low IgG/IgM ratio [68]. Generation of highly avid IgG antibodies to the cell surface antigens could therefore be a target for an effective vaccine or immunotherapy [69]. Also, given the complexity of disease pathogenesis, heterogeneity of the human population and their immune responses, a judicious mix of Abs against selected antigens may be desirable.

## Supplementary Material

Supplemental
