# Supplementary material for: Naturally produced opsonizing antibodies restrict the survival of Mycobacterium tuberculosis in human macrophages by augmenting phagosome maturation
Source: Open Biol. 2015 Dec 16;5(12):150171. doi: 10.1098/rsob.150171 (PMC4703058; doi:10.1098/rsob.150171)
Supplement: Supplemental [file rsob150171supp1.docx]

**Supplemental figure S1**


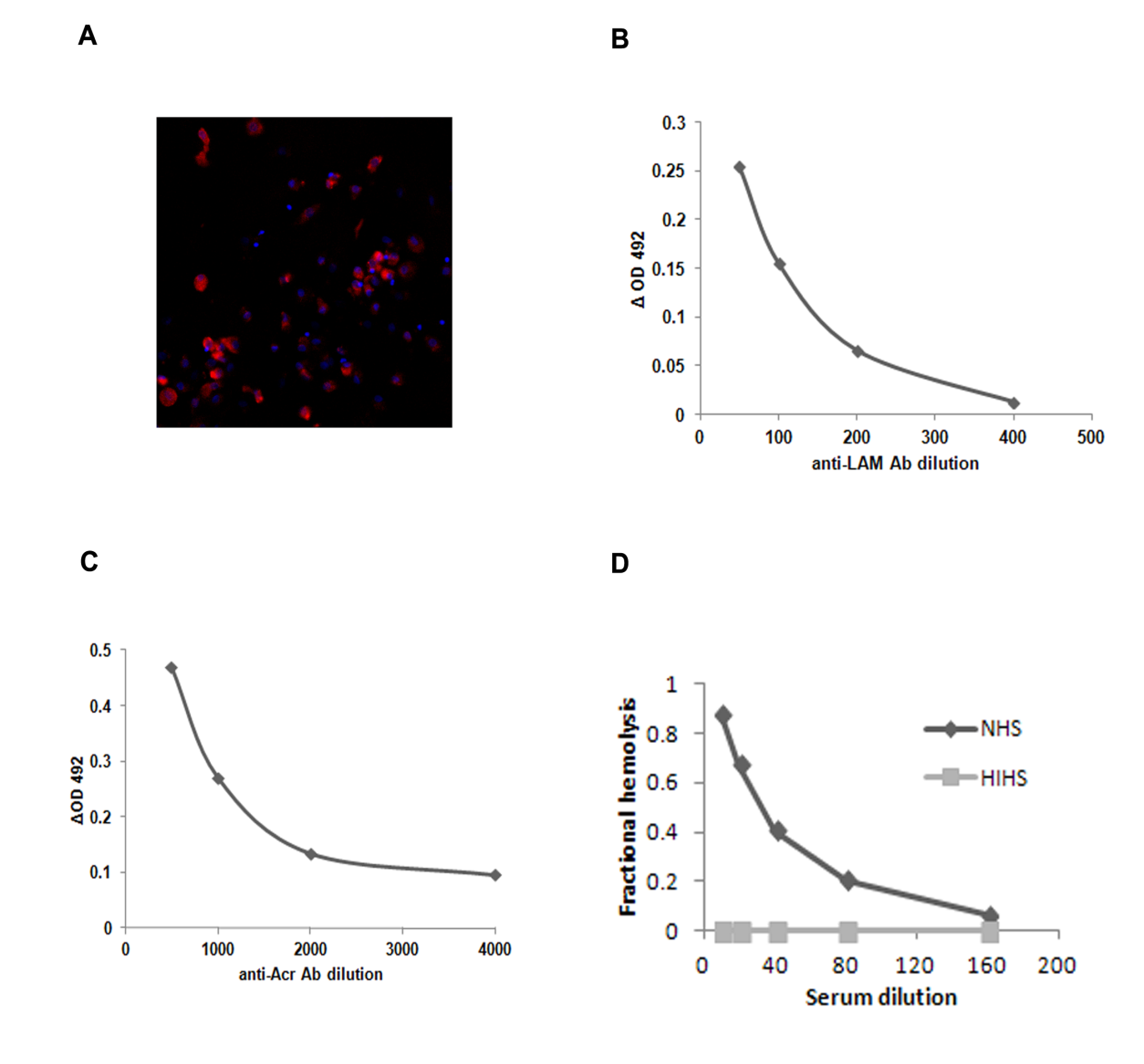


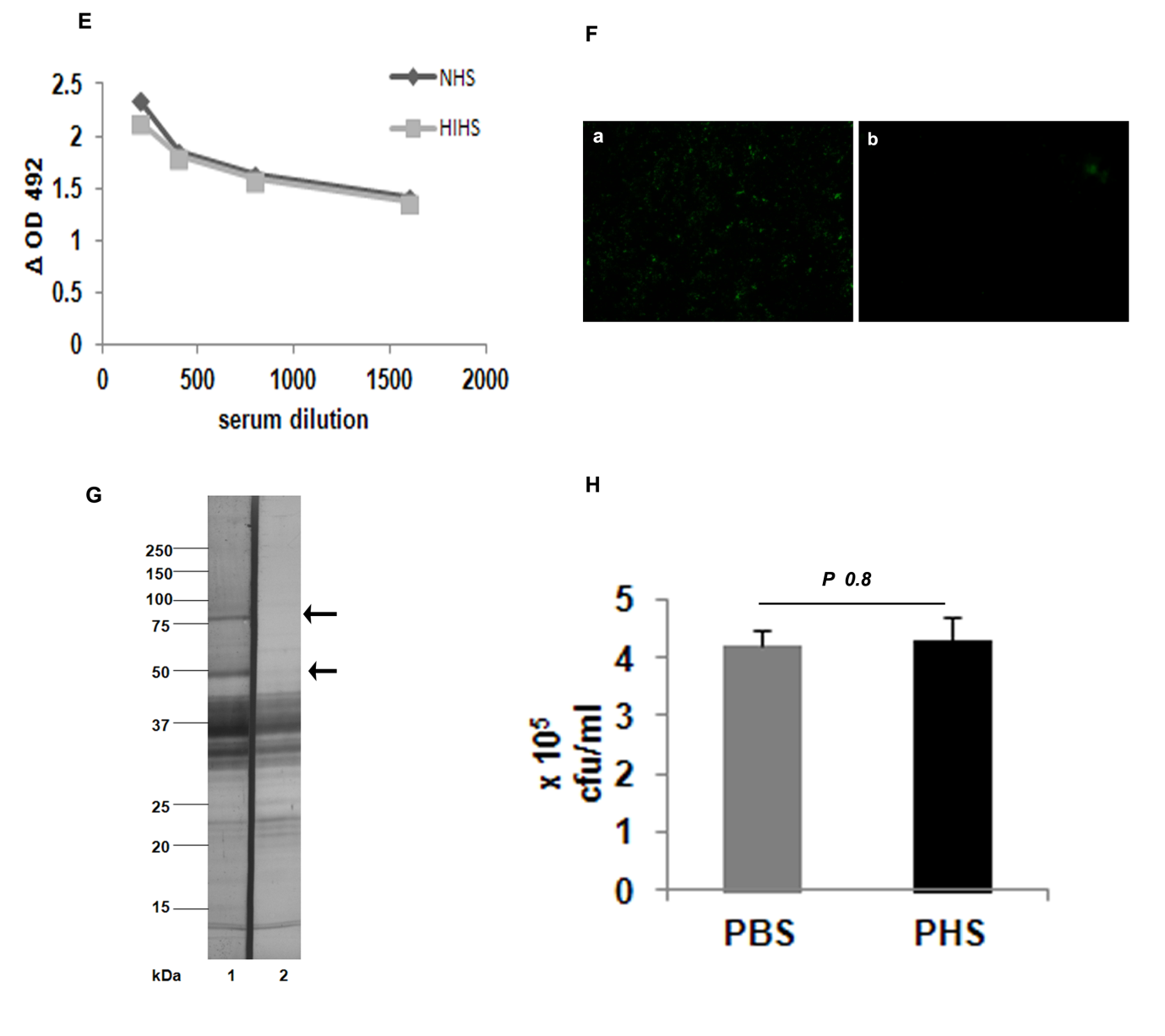


**Supplemental figure S2**


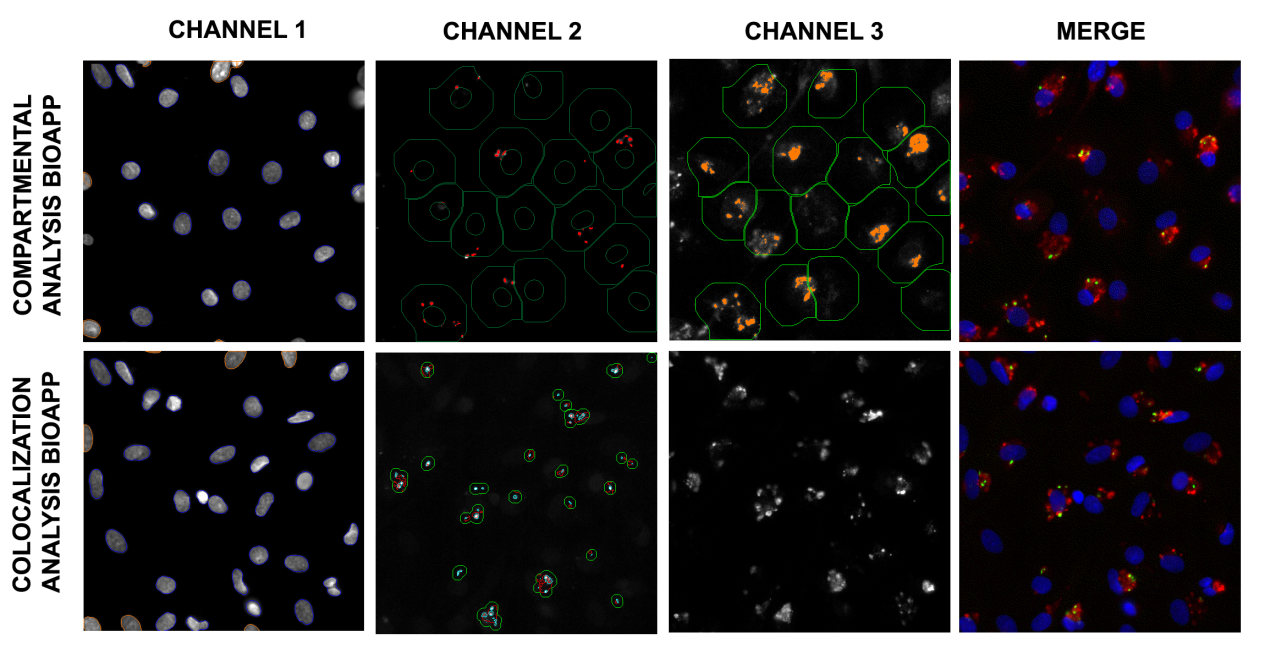


**Supplemental figure Legends**

**Fig. S1A**. Expression of CD14 marker protein on the surface of adherent monocytes.

**Fig. S1B.** Concentration-dependent bindings of anti-LAM (CS-35) mAb to Mtb cell membrane, determined by ELISA.

**Fig. S1C.** Concentration-dependent bindings of anti-Acr (IT-4) mAb to Mtb cell membrane, determined by ELISA.

**Fig. S1D.** Heat-inactivation of sera resulted in depletion of hemolytic complement. Sheep RBC fractional haemolysis values for normal (NHS) and heat-inactivated serum (HIS).

**Fig. S1E.** Heat-inactivation of sera did not affect antibody titres against cell membrane antigens**.** Ab (IgG) titres in NHS and HIS were determined by ELISA.

**Fig. S1F.** Opsonization with heat-inactivated serum resulted in binding of IgG class of antibodies to Mtb cell surface. Fluorescent micrographs of **a.** Opsonized and **b.** Unopsonized Mtb are shown.

**Fig. S1G.** Immunobloting of membrane antigens with pooled donor serum (lane-1) and Abs extracted from the opsonized Mtb (lane-2). Antibodies to at least two major antigens (of approximately 48 and 80 kDa, marked with arrows) did not participate in opsonization.

**Fig. S1H.** Opsonization with heat-inactivated serum had no adverse effect on cell viability of Mtb. Bar graph represents cfus of opsonized (with heat-inactivated serum) and unopsonized (incubated with PBS) bacilli.

**Fig. S2.** Schematic of image analysis using Cellomics Compartmental Analysis and Colocalization Analysis Bioapplication.
